# Nanostructural Analysis of Enzymatic and Non-enzymatic Brown Rot Fungal Deconstruction of the Lignocellulose Cell Wall^†^

**DOI:** 10.3389/fmicb.2020.01389

**Published:** 2020-06-24

**Authors:** Yuan Zhu, Nayomi Plaza, Yuka Kojima, Makoto Yoshida, Jiwei Zhang, Jody Jellison, Sai Venkatesh Pingali, Hugh O’Neill, Barry Goodell

**Affiliations:** ^1^School of Materials Science and Engineering, Central South University of Forestry and Technology, Changsha, China; ^2^Forest Products Laboratory, USDA Forest Service, Madison, WI, United States; ^3^Department of Environmental and Natural Resource Science, Tokyo University of Agriculture and Technology, Fuchu, Japan; ^4^Department of Bioproducts and Biosystems Engineering, University of Minnesota, Saint Paul, MN, United States; ^5^Center for Agriculture, Food and the Environment, University of Massachusetts, Amherst, MA, United States; ^6^Biology and Soft Matter Division, Oak Ridge National Laboratory, Oak Ridge, TN, United States; ^7^Department of Microbiology, Morrill Science Center IV-N, University of Massachusetts, Amherst, MA, United States

**Keywords:** brown rot of lignocellulose, wood decay fungi, non-enzymatic activity, LPMO upregulated enzyme expression, chelator-mediated Fenton (CMF) degradation, biorefinery, CAZymes, SANS (small-angle neutron scattering)

## Abstract

Brown rot (BR) decay mechanisms employ carbohydrate-active enzymes (CAZymes) as well as a unique non-enzymatic chelator-mediated Fenton (CMF) chemistry to deconstruct lignocellulosic materials. Unlike white rot fungi, BR fungi lack peroxidases for lignin deconstruction, and also lack some endoglucanase/cellobiohydrolase activities. The role that the CMF mechanism plays in “opening up” the wood cell wall structure in advance of enzymatic action, and any interaction between CMF constituents and the selective CAZyme suite that BRs possess, is still unclear. Expression patterns for CMF redox metabolites and lytic polysaccharide monooxygenase (LPMO–AA9 family) genes showed that some LPMO isozymes were upregulated with genes associated with CMF at early stages of brown rot by *Gloeophyllum trabeum*. In the structural studies, wood decayed by the *G. trabeum* was compared to CMF-treated wood, or CMF-treated wood followed by treatment with either the early-upregulated LPMO or a commercial CAZyme cocktail. Structural modification of decayed/treated wood was characterized using small angle neutron scattering. CMF treatment produced neutron scattering patterns similar to that of the BR decay indicating that both systems enlarged the nanopore structure of wood cell walls to permit enzyme access. Enzymatic deconstruction of cellulose or lignin in raw wood samples was not achieved via CAZyme cocktail or LPMO enzyme action alone. CMF treatment resulted in depolymerization of crystalline cellulose as attack progressed from the outer regions of individual crystallites. Multiple pulses of CMF treatment on raw wood showed a progressive increase in the spacing between the cellulose elementary fibrils (EFs), indicating the CMF eroded the matrix outside the EF bundles, leading to less tightly packed EFs. Peracetic acid delignification treatment enhanced subsequent CMF treatment effects, and allowed both enzyme systems to further increase spacing of the EFs. Moreover, even after a single pulse of CMF treatment, both enzymes were apparently able to penetrate the cell wall to further increase EF spacing. The data suggest the potential for the early-upregulated LPMO enzyme to work in association with CMF chemistry, suggesting that *G. trabeum* may have adopted mechanisms to integrate non-enzymatic and enzymatic chemistries together during early stages of brown rot decay.

## Introduction

Nature has developed various systems for wood degradation (Eastwood et al., 2011; Cragg et al., 2015; Daniel, 2016; Goodell, 2020). In general, biomass-degrading fungi rely on complex degradative machineries that basically catalyze two types of processes: (1) generation of oxidative species (e.g., radicals) that act directly on the biomass; and (2) direct enzymatic depolymerization, for example by glycoside hydrolases. In most white rot fungi, the mode of attack is primarily enzymatic, but cell wall-degrading enzymes are too large to penetrate the intact wood cell wall, so in simultaneous white rots, erosion of the cell wall proceeds only from exposed lignocellulose surfaces. Brown rot (BR) fungi have been recognized as evolving from the predecessors of current white rot fungi and, as part of this evolutionary process, lignolytic enzyme systems and crucial types of cellulases in BRs have been lost (Hibbett and Donoghue, 2001; Floudas et al., 2012). In BRs, a chelator-mediated Fenton (CMF) system has evolved to replace at least some of the cellulolytic enzymatic machinery possessed by progenitor white rot fungi. The replacement of physiologically expensive extracellular enzymes with a low-molecular weight system that permits the efficient deconstruction of both holocellulose and lignin has been considered to confer an evolutionary advantage to brown rot fungi in exploiting some types of woody tissue (Goodell et al., 1997; Eastwood et al., 2011; Arantes et al., 2012; Floudas et al., 2012).

Efficiency is gained in brown rot attack because low molecular weight metabolites can diffuse deep within the wood cell wall to catalyze depolymerization at more internal cell wall sites than are accessible to larger enzymes (Goodell, 2020). The CMF system is unique among all biological systems in being the only substrate deconstruction system known based on oxygen radical chemistry that permits non-enzymatic deconstruction at a considerable distance (several microns) from the organism (Goodell et al., 2014). Generation of an oxidative mechanism deep within the wood cell wall, and apart from the fungal hyphae, is important for a number of reasons not the least of which is that, generation of an indiscriminate ROS oxidative mechanism must occur in a manner that does not cause damage either to the fungus or to the extracellular enzymes secreted by the fungus. Prior research has demonstrated that many extracellular hydrolytic carbohydrate-active enzymes (CAZymes) are expressed in later stages of BR decay (delayed temporal expression) after the cell wall structure has been non-enzymatically modified to permit enzyme access (Zhang et al., 2016, 2019; Presley and Schilling, 2017). Although this has been discussed as a mechanism to avoid ROS damage to enzymes, our research has also demonstrated that because of differing micro-environmental conditions, pH shifts, and other parameters, ROS generation by CMF action would occur only within the wood cell wall, which is inaccessible to enzymes (Goodell et al., 1997; Arantes and Goodell, 2014). Therefore, we propose that delayed temporal expression of most CAZymes in brown rot fungi is due primarily to the fact that secretion of most enzymes at early decay stages would not result in depolymerization of the cell wall. Instead, the delayed expression of CAZymes permits these enzymes to have maximal effectiveness after the cell wall structure has been opened by non-enzymatic CMF action, in a type of energy investment strategy to permit CAZyme digestion of holocellulose within the cell walls at this later stage (Zhang et al., 2019).

The brown rot non-enzymatic CMF mechanism has been explored only in a limited manner for use in biomimetic industrial/biorefinery applications (Goodell et al., 2017; Kent et al., 2018; Liu et al., 2020). Prior work with Fenton chemistry alone has in many cases not taken into account the short half-life of hydroxyl radicals generated. Thus, in some cases, Fenton-type treatments for industrial processing have been designed without an understanding that reactants must be allowed to sequentially penetrate and then react within the cell wall to efficiently generate hydroxyl radicals where needed for cell wall depolymerization (Goodell et al., 2014). In our previous work, results show that the CMF-iron treatment was highly efficient in opening up the pore structure of wood, and indicate that a single-stage treatment modifies wood in a manner similar to that achieved by a moderate level of decay by brown rot fungi. However, fungal metabolism of sugars does not occur with CMF treatment, and the resulting sugars and oligosaccharides produced can potentially be used in downstream fermentation or other industrial processes (Goodell et al., 2017). Furfurals and hydroxymethyl furfurals are typically not generated using Fenton chemistries with biomass substrates unless high heat is used, and further, Fenton chemistries have been used to reduce the amount of furfurals generated in biomass deconstruction (Yang et al., 2014, 2019).

Lytic polysaccharide monooxygenase (LPMO) enzymes are known to be the only redox enzyme which can depolymerize crystalline cellulose in delignified cell walls directly. LPMOs previously had been considered to use oxygen as an electron acceptor in the catalytic reactions; however, in more recent studies hydrogen peroxide, which is also well-known as a component of the CMF system, has been recognized to be a more efficient electron acceptor with the catalytic efficiency of LPMO with hydrogen peroxide being significantly higher than that with molecular oxygen (Bissaro et al., 2017; Forsberg et al., 2019). Although research on the actual role of LPMO in lignified wood has been limited, high molecular weight (HMW) lignin and low molecular weight (LMW) lignin have been reported to serve as electron donors for LPMO9 (Muraleedharan et al., 2018), resulting in enhanced LPMO efficiency in cellulose deconstruction. Because CMF chemistry fragments lignin (Goodell et al., 2017), and this lignin also serves to donate electrons which reduce iron (Tamaru et al., 2019), this suggests that *G. trabeum* LPMO activity might be enhanced by CMF activity in a manner not yet understood. The manner in which the CMF mechanism may “open up” the structure of wood cell walls in advance of enzymatic action also is unknown, as is the mode of LPMO action on lignocellulose either with or without CMF treatment.

In the current study, we explored potential pathways for the biosynthesis of iron-reducing chelators produced by *G. trabeum* involved in CMF chemistry. This mechanistic expression analysis was done with the aim of helping to integrate our current understanding of both non-enzymatic and enzymatic brown rot decay mechanisms with nanostructural data and to characterize changes that occur when wood is treated with various systems to mimic brown rot decay processes. We also then conducted a nanostructural analysis, where experiments with the CMF system modeled using different levels of treatment were conducted, and we compared treatments with and without CAZyme treatment. In some samples, peracetic acid was used to remove lignin prior to treatment and permit better understanding of attack on the cellulose structure. These treatments were used as baseline, or reference treatments, for comparison to the CMF and enzyme treatments and compared to wood decayed by *G. trabeum*. Additional undecayed wood samples were treated using either CMF chemistry, or CMF chemistry followed by treatment with either a recombinant AA9-LPMO cloned from *G. trabeum* (GtLPMO9A-2), which was reported to show activity not only for cellulose but also for some hemicelluloses such as xyloglucan and glucomannan (Kojima et al., 2016), or by a CAZyme cocktail (a commercial blend of cellulases/xylanases). Modification of the structure of wood cell walls was characterized using small angle neutron scattering (SANS). Enhancing understanding of brown rot degradative mechanisms will lead to better solutions to address recalcitrance problems associated with lignin encrustation of cellulose in the pretreatment of biomass in biorefinery applications.

## Materials and Methods

**FIGURE 1:**
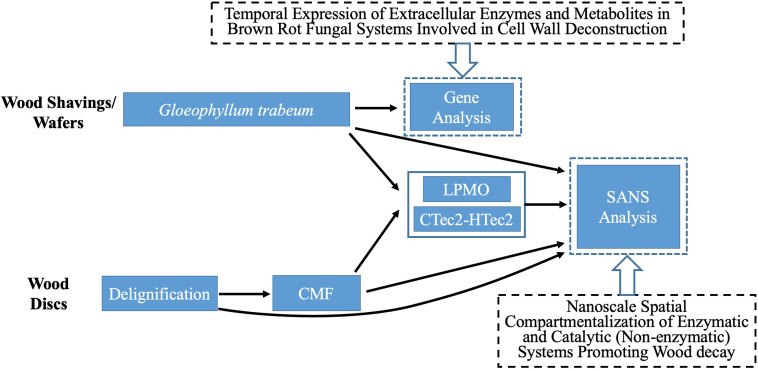
Flow chart of materials, treatments and analyses used in our procedures.

Figure 1 provides a schematic summary of the procedures used in this research.

### Gene Expression Patterns

To explore the interaction between LPMO and CMF in terms of expression timing, gene expression levels were retrieved from RNA-seq datasets obtained by measuring mRNA levels from *G. trabeum* in decayed thin aspen wood wafers (Gene Expression Omnibus number GSE108189) (Zhang et al., 2019). In this work, a ‘thin wood wafer design’ was used to promote fungal growth along a strip of wood, which was then sequentially sectioned to allow the different decay stages to be assessed using temporal transcriptomics for brown rot decay (Zhang et al., 2016). Specifically an American Society for Testing and Materials soil microcosm chamber was used to culture *G. trabeum* (Zhang et al., 2016). The soil medium was composed of a 1:1:1 mixture of soil, peat, and vermiculite, hydrated to 40–45% (wt/vol) moisture content as specified in the standard. Sterilized aspen (*Populus* sp.) wood wafers (2.5 mm thick × 25 mm wide × 60 mm long) were placed at an angle in the microcosm chambers with the 25 mm edge resting on the soil surface, for colonization from the base by hyphae. Wafers were harvested after the advancing hyphal front had colonized 50 mm up the length of a wafer. The wafers were then sectioned into 5-mm sections along their entire length, using advancing hyphal front as the starting point (0 mm), and short wafer sections the cut from the 0–5 mm, 15–20 mm and 30–35 mm zones. These zones were then sampled for transcriptomic analysis, representing early-, mid-, and late-decay stages of brown rot, respectively.

Expression levels in Reads Per Kilobase of transcript per Million mapped (RPKM) of genes relating to hydroquinone, oxalate and LPMO synthesis were studied for their temporal expression patterns, as brown rot decay by *G. trabeum* progressed. A key metabolite family of interest in early stages of BR wood decay is the low molecular weight (LMW) hydroquinone family of metabolites including 2,5-dimethoxyhydroquinone (DMHQ) and 4,5-dimethoxy-1,2-benzenediol (DMC) (Goodell et al., 1997; Kerem et al., 1999; Paszczynski et al., 1999).

A *de novo* synthesis pathway was built in brown rot fungi according to previous studies on aromatic metabolites in wood-decaying fungi, and the key pathway genes involved in hydroquinone synthesis were identified via a BlastP search of the relevant homologs in the *G. trabeum* genome (Hattori et al., 1999; Lapadatescu et al., 2000; Meganathan, 2001).

### Nanostructural Characterization

#### Wood Samples

Southern yellow pine (SYP - *Pinus* spp.) sapwood wood shavings (110–160 μm thick) were prepared using a commercial wood-shop planer. Alternately, SYP sapwood disks (19 mm diameter, 0.5 mm ∼ 0.6 mm thick) were cut using a sledge microtome. Samples are referred to as “shavings” or “disks” in this research. Disks were used for chemical (CMF), delignification and enzyme treatments. Shavings were used for decay treatments because disks lost structural integrity rapidly in decay tests and it was not possible to transfer them into the SANS sample holders for analysis.

#### Peracetic Acid (PAA) – Delignification Treatments

PAA solution (30% wt/wt) was prepared using deionized (DI) water, and 1 g of air-dried SYP wood disks were saturated with 10 mL PAA solution in a shaker bath at 100 rpm and 25°C for 24 h (Chang and Holtzapple, 2000). Delignified SYP disks were then washed five times each with 40 ml DI water, and dried at 60°C for 24 h. PAA treatment and washing were repeated 3X to remove lignin. Mass loss for all samples averaged 29 ± 5% after 3 PAA treatment cycles. No shaving samples were treated with PAA.

#### Chelator-Mediated Fenton (CMF) Treatments

For CMF treatment, three replicate wood disks each, with/without PAA-delignification, were treated with 50 mM iron (III) chloride hexahydrate (FeCl_3_.6H_2_O) in acetate buffer (pH 4.0, 1M) at a loading of 0.2 g wood/5 ml buffered iron (III) solution. Sample materials were thoroughly mixed for 10 min to allow the iron to penetrate and bind to cellulose deep within the wood cell wall, and then dried at 120°C for 2 h to remove moisture prior to the addition of the redox chelator. 2,3-dihydroxybenzoic acid (2, 3-DHBA) 50 mM was then added as the redox chelator at 25°C with shaking for 30 min using an equal volume to the initial iron (III) solution. The same volume of H_2_O_2_ (final molarity of 0.5 M) was then added with hand mixing for 5 min followed by water bath shaking (125 rpm) at 40°C overnight. Samples were then drained of all solutions, and another fresh batch of 0.5 M H_2_O_2_ was added again. After 24 h of incubation (25°C, 125 rpm), the H_2_O_2_ was drained from the samples. These procedures were repeated 2X or 4X (designated as 2x or 4x pulses) to produce the CMF treated samples (Table 1), which were stored frozen for additional treatment or further analysis. Note: PAA-delignified wood disks were unable to go through 4-pulses of CMF treatment as the wood was solubilized at that point.

**TABLE 1:** Mass losses comparing unmodified wood or wood that was PAA-delignified and then treated with either 1, 2, or 4 pulses of the CMF system.

Samples	CMF	Mass loss
Unmodified Wood	1-pulse	4%
	2-pulses	16%
	4-pulses	78%
Delignified wood	1-pulse	33%
	2-pulses	36%

*Only single samples were available for the mass loss measurements.*

#### Decay Treatment

Decay treatment was the same as that described previously (Goodell et al., 2017). Wood shavings were incubated with mycelium from liquid cultures of *G. trabeum* for either 18 or 42 days (2 replicates each), designated as 18dGt and 42dGt, respectively. After incubation, samples were carefully transferred into the titanium cells for SANS analysis.

#### Enzyme Treatment

A recombinant LPMO (GtLPMO9A-2) was cloned from *G. trabeum*, and heterologously expressed in the yeast *Pichia pastoris* as detailed previously (Kojima et al., 2016). This enzyme has activity on cellulose, carboxymethylcellulose and broad activity on hemicelluloses such as glucomannan and xyloglucans (Kojima et al., 2016). The use of the LPMO on samples in this research was compared to use of a combination of commercial enzymes (CTec2 and HTec2 – Novozymes, Bagsvaerd, Denmark) mixed in a cocktail.

LPMO treatments were applied at a rate of 1 μM (final concentration), and the CTec2 and HTec2 mixture (at a ratio of 4:1) was applied at a final concentration 10-12FPU/g wood in 50 mM acetate buffer (pH 5). The activity of the CTec2 and HTec2 enzymes was assessed prior to use via a filter paper activity assay (Bailey, 1981). Enzymes were used to treat wood disks or wood shavings either without pretreatment (reference treatments) or after samples were pretreated using the PAA treatment for delignification, CMF treatment, or by decay with *G. trabeum* as detailed in Table 2. All enzyme treatments were conducted in a water bath shaker for 72 h (45°C, 100 rpm). Samples were then subsequently washed with DI water before oven-drying.

**TABLE 2:** Treatments of all wood samples analyzed by SANS.

Labels	PAA delignification	CMF	*G. trabeum*	Enzyme
UW	–	–	–	–
UW-CMF-1	–	+	–	–
UW-CMF-2	–	++	–	–
UW-CMF-4	–	++++	–	–
UW-LPMO	–	–	–	LPMO
UW-CMF-1-LPMO	–	+	–	LPMO
UW-CMF-2-LPMO	–	++	–	LPMO
UW-CTec2-HTec2	–	–	–	CTec2-HTec2
UW-CMF-1-CTec2-HTec2	–	+	–	CTec2-HTec2
UW-CMF-2-CTec2-HTec2	–	++	–	CTec2-HTec2
0 dGt	–	–	–	–
18 dGt	–	–	18 dGt	–
42 dGt	–	–	42 dGt	–
18 dGt-LPMO	–	–	18 dGt	LPMO
42 dGt-LPMO	–	–	42 dGt	LPMO
18 dGt-CTec2-HTec2	–	–	18 dGt	CTec2-HTec2
42 dGt-CTec2-HTec2	–	–	42 dGt	CTec2-HTec2
DW-CMF-1	+	+	–	–
DW-CMF-2	+	++	–	–
DW-LPMO	+	–	–	LPMO
DW-CTec2-HTec2	+	–	–	CTec2-HTec2

*For CMF-treated wood: +, single pulse; ++, double pulse; ++++, 4-pulse CMF treatment.*

#### SANS Analysis

##### Terminology

For appropriate interpretation, it is helpful to define the following terms used in SANS data analysis. These definitions are very brief, appropriate for this research, and they are not intended to provide a detailed understanding of SANS data analysis:

###### Low-q, mid-q, and high-q

These terms represent regions of the *x*-axis (scattering vector q) of the SANS curves which are associated with the size of features in the sample contributing to the scattering. Low-q extends from 0.001 – 0.01 [Å^–1^], mid-q from 0.01 – 0.1 [Å^–1^], and high-q from 0.1–0.3 [Å^–1^].

###### Power Law Exponent

This is a number that describes the shape of the different sections of the SANS curves; specifically, the low-q and mid-q sections. In our research, the shape of the SANS curves varies as changes occur in the samples, and the mid-q Power Law Exponent generally increases as separation of EFs, and other angstrom and nanoscale separations, occur.

###### Radius of gyration (*R*_g_)

This term defines the average characteristic size of a nanoscale feature as the average radius/distance from the center of mass of the feature taking into account aspect ratio. Here, the *R*_g_ may correspond to a space or a gap which may change in size with treatments, but it also may show how the size of polymeric agglomerates (repolymerized lignin fragments) may be changing in response to treatments.

##### Methodology

**FIGURE 2:**
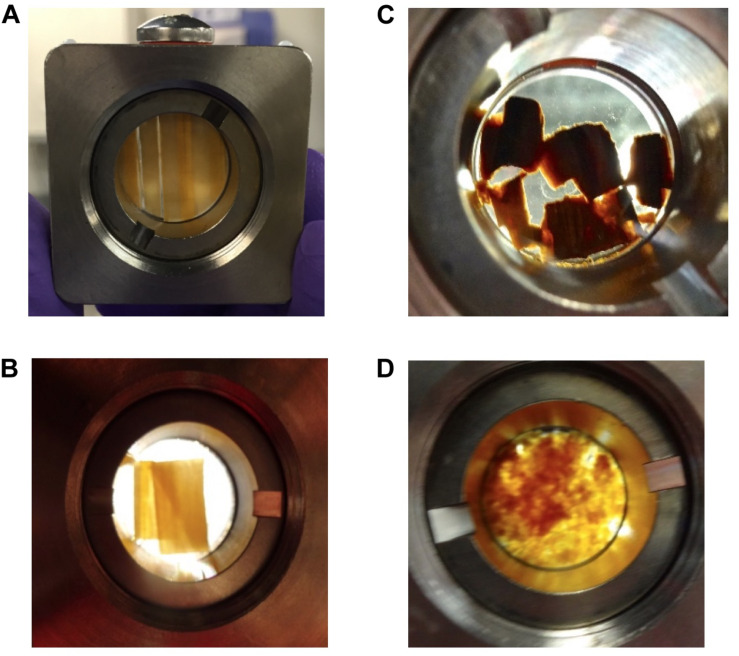
D_2_O saturated samples loaded into titanium cells for SANS analysis showing the differences in sample alignment inside those cells. The majority of the **(A)** wood disks and **(B)** wood shavings were aligned vertically inside the titanium cells, whereas a few of the **(C)** delignified disks were less well oriented and the **(D)** decayed wood shavings were randomly oriented.

After being treated as described in Table 2, D_2_O saturated samples were loaded into titanium cells, with quartz windows and a 0.5 mm aluminum spacer. Once the titanium holder was screw-sealed, it was filled with D_2_O and all air bubbles were removed. To the extent possible, both disks and shavings were aligned inside the titanium cells so that the wood grain was oriented vertically (Figures 2A,B). Overlapping and/or misalignments inside the titanium cells were observed for some of the delignified wood disks and wood shavings (Figures 2C,D). Misalignments inside the wood cell wall likely decreased the sharpness of the diffraction peak in the aligned scattering from these samples, consequently, increasing the polydispersity and uncertainty in the measured values of the elementary fibril spacing of samples exposed to more severe treatment like DW-CMF-2 or DW-LPMO.

**FIGURE 3:**
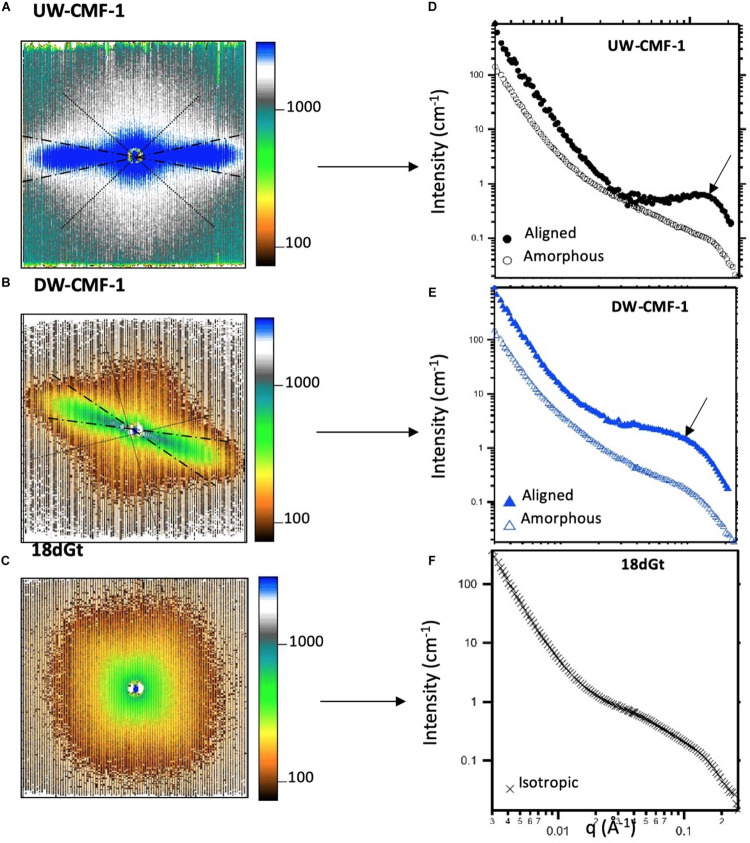
Uncorrected SANS 2D detector images from **(A)** an unmodified wood disk treated with one pulse of CMF, **(B)** a delignified wood disk treated with one pulse of CMF, and **(C)** wood shavings decayed by *G. trabeum* for 18 days. The aligned sectors used in the data reduction are shown between the dashed radians, and the amorphous sector is enclosed within the dotted lines. All patterns were measured at a sample-to-detector distance of 1.7 m. **(D–F)** Scattering profiles reduced from the anisotropic and isotropic scattering patterns showing the aligned (closed symbols), amorphous (open symbols) and isotropic curves (crosses). Arrows in d and e show the location where the diffraction peak is observed.

SANS measurements were performed on the Bio-SANS beam line at the High Flux Isotope Reactor facility in the Oak Ridge National Laboratory (Oak Ridge, TN, United States). All scattering patterns were acquired using a neutron wavelength λ of 6 Å, with a wavelength spread (Δλ/λ) of 13%. To achieve a broad scattering vector range (from 0.003 to 0.27 Å^–1^) two sample-to-detector distances (1.7 and 14.5 m) were necessary. The shorter sample-to-detector distance provided the higher q data, and the longer distance was used to acquire the lowest *q* values. Circular aperture diameters of 40 and 14 mm were used for the source and sample, respectively. The radial distance from the beam-center to the edge of the detector images was converted into the reciprocal space scattering vector q, which describes the relationship between the neutron wavelength λ and the scattering angle θ as *q* = 4πsin(θ)/λ. The detector images were normalized to the incident beam monitor counts, and corrected for pixel sensitivity, dark current and background contributions. Then, the images were transformed into polar reciprocal space to reduce the data anisotropically into two profiles: aligned and amorphous. If no anisotropy was observed (Figure 3C), then the image was reduced isotropically into one scattering curve. Features with strong preferential orientation such as the regularly packed cellulose elementary fibrils (EFs) in the S2 cell wall layer contributed to the anisotropic scattering in the aligned sector (Figure 3A), whereas scattering from all the lesser organized wood polymers contributed to the isotropic or amorphous scattering. This reduction was implemented using the IgorPro macros provided at the beamline. Figure 3 shows the typical scattering patterns observed in this study, and the corresponding 1D scattering profiles obtained from the data reduction.

SANS 1D profiles were analyzed using the IRENA package implemented in IgorPro (Ilavsky and Jemian, 2009). Based on the different structural features observed in our study, we fitted the data using an empirical model consisting of a low-q power law, a mid-q unified fit level and a Gaussian peak. While several models have been used to fit scattering data from wood, including (a) empirical models (Fernandes et al., 2011; Thomas et al., 2014; Plaza et al., 2016), (b) variations of the Unified fit (Goodell et al., 2017), and (c) cylinder-based models (Jakob et al., 1996; Penttila et al., 2019), in our current research we chose an empirical model in order to detect the changes caused by the various treatments without making assumptions about the cellulose microfibril arrangement in the wood cell wall. Some of our treatments caused lignin to be modified, and a model appropriate for changes which occur in both lignin and cellulose must be considered. However, even though the empirical model was the best suited for analyzing our work, the trends reported are not model-dependent and to the best of our knowledge, would not change if a different model was used to fit the data.

For the wood disks, which mostly scattered anisotropically, the peak was enhanced by subtracting the amorphous profiles from the aligned curves, which removed the mid-q scattering contribution. These subtracted curves were fitted with only two structural levels to extract the low-q power law (P_1_) and the peak position (q_0_), which has been used to measure the spacing between elementary fibrils (EFs) in wood using the following equation 2π/q_0_ (Fernandes et al., 2011; Thomas et al., 2014; Plaza et al., 2016). Then, the amorphous profiles were fitted using the model with three structural levels to extract the mid-q radius of gyration (R_g_) and power law exponent (P_2_). For the wood shavings, which scattered isotropically, the reduced scattering profiles exhibited weak features, and the data were fitted using the model with the three structural levels, though for these samples, differences were only observed in the mid-q scattering region. All uncertainties reported reflect both the natural variability and polydispersity of the structural features in wood, as well as the goodness of the fit, which in our case was calculated in IRENA as the range satisfied by this condition: $\chi_{f\,i\,t}^{2}<1.06\,\chi_{c\,r\,i\,t\,i\,c\,a\,l}^{2}$. It should be noted that SANS is a bulk measurement technique and thus, while each scattering profile is obtained from a single sample, all structural features within the accessible length scale range contribute to the measured profile.

## Results and Discussion

### Hypothesized Gene Expression Pathways for DMHQ and Related LMW Redox Metabolites, With Proposed Interaction Between CMF and LPMO Genes

LMW metabolites play important roles in CMF chemistry, among which catechol/hydroquinone derivatives (Goodell et al., 1997; Paszczynski et al., 1999) (e.g., 2,5-dimethoxy-1,4-benzenediol [hydroquinone] – DMHQ and 4,5-dimethoxy-1,2-benzenediol – DMC) (Kerem et al., 1999) and related LMW compounds (Eastwood et al., 2011) (e.g., variegatic acid) have been most commonly reported. The ability of these compounds to reduce iron to Fe^2+^ has been validated by several groups, and H_2_O_2_ produced through redox cycling has also been proposed (Varela and Tien, 2003). At least three independent brown rot lineages are known to produce these LMW catecholate/hydroquinones and related iron-reducing compounds (Floudas et al., 2012; Eastwood, 2014), which are here referred to simply as “chelators.”

**FIGURE 4:**
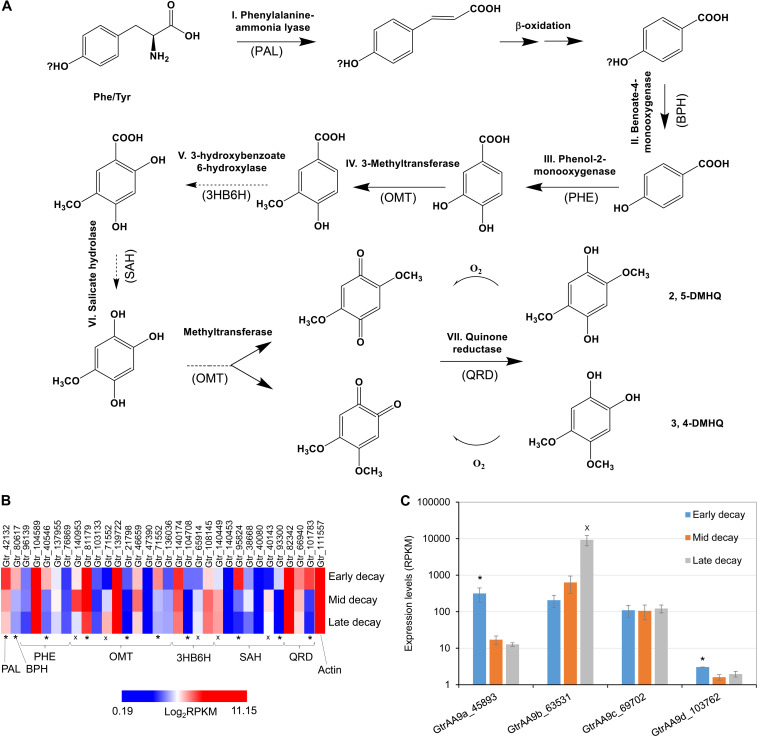
Co-expression of LPMO and LMW hydroquinone pathways at an early brown rot stage in *G. trabeum*. **(A)** The hydroquinone chelator synthesis pathway and the enzyme homologs catalyzing each step are proposed based on standard pathways. Genes that were significantly upregulated (*, FDR < 0.05 by RNA-seq) in early brown rot basically covered all the required key enzyme components of the pathway, although several OMT, 3HB6H and SAH genes were clearly upregulated at late decay stages (x, FDR < 0.05) **(B)**. Expression timing of *G. trabeum* LPMO genes showed that AA9a was significantly upregulated in early brown rot stages, and these were co-expressed with genes for metabolites produced in proposed pathway for hydroquinone synthesis. **(C)** Expression levels for GtrAA9 genes during early-, mid- and late-decay stages.

In this research we found that the genes associated with hydroquinone chelator synthesis pathways were more likely upregulated at early brown rot stages in *G. trabeum* (Figures 4A,B), indicating the roles of these metabolites in non-enzymatic cell wall deconstruction during the incipient stage of brown rot. This is in line with our previous report that showed several genes encoding LMW metabolites were upregulated early during brown rot. Our previous data also showed that most of the CAZymes, including many cellulases, were upregulated late and the expression pattern for AA9b (JGI protein ID 63531; Figure 4C) is in agreement with this. Conversely however, two LPMO genes AA9a (45893, designated GtLPMO9A) and AA9d (103762, designated GtLPMO9D) were found to be upregulated significantly at early decay stages. GtLPMO9A-2, which is one of the splicing variants transcribed from the AA9a gene, was reported to show activity not only against cellulosic substrates but also against hemicellulosic substrates such as glucomannan (Kojima et al., 2016), which is one of the primary hemicelluloses in softwood. Considering the relatively high expression levels of the AA9a gene at an early decay stage (Figure 4C), we propose that this enzyme may play an important role in the degradation of the wood cell wall ahead of hydrolytic enzymes such as cellulases. Moreover, co-expression of the AA9a gene with the genes involved in the synthesis of hydroquinones indicates that GtLPMO9A might potentially work cooperatively with hydroquinones in the reaction process of the non-enzymatic chelators produced by *G. trabeum* and associated with CMF chemistry. There have been no prior reports on the enzymatic characteristics of GtLPMO9D, but we found very low expression levels of the AA9d gene (RPKM < 10) suggesting perhaps only limited association of the GtLPMO9D enzyme with LMW chelators.

We also observed that several catechol *o*-methyltransferase (OMT), 3-hydroxybenzoate 6-hydroxylase (3HB6H), and *s*-adenosyl-l-homocysteine (SAH) genes were upregulated in late decay stages (Figure 4B). We propose that this is related to detoxification and polymerization of low levels of soluble lignin which would have been released into the extracellular fungal matrix (ECM) which surrounds the fungal hyphae, as the lignin was being initially depolymerized by CMF attack during decay. OMT and 3HB6H can act on phenolic fragments to modify and detoxify them, while SAH prevents methylation of phenolics. Preventing methylation of lignin monomer fragments (by *S*-adenosyl methionine) potentially would promote additional repolymerization of lignin radical monomers which would be consistent with the nature of brown rotted lignin.

### Nanostructural Characterization

#### Effects of Pulsed CMF Treatments and G. *trabeum* Decay on Wood Cell Walls

**FIGURE 5:**
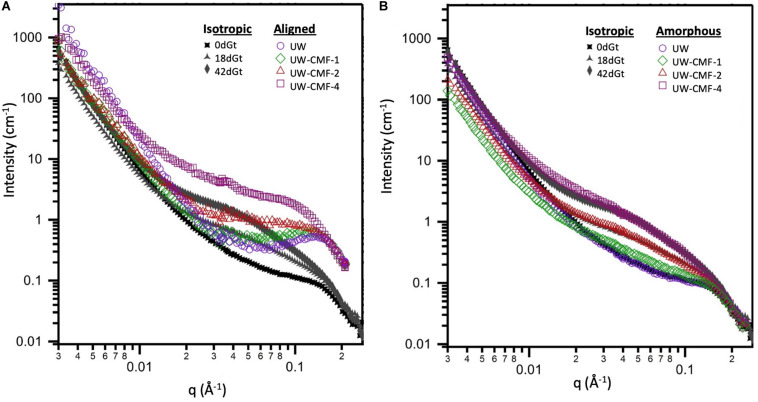
Comparison of the effect of CMF treatment on sound wood, and decay by *G. trabeum*, on the wood’s nanostructure. Both **(A,B)** plots show the isotropic scattering profiles of the decayed samples which are plotted together with either: **(A)** the aligned scattering curves from the pulsed CMF-treated samples or **(B)** the amorphous scattering profiles from the pulsed CMF-treated samples.

Two-dimensional scattering patterns from the CMF treated disks were anisotropic, and featured strong, aligned scattering sectors. Whereas all decay treated shaving samples exhibited isotropic scattering, due to their random orientation inside the cell. Reducing the patterns into one-dimensional profiles allowed us to compare the effects of the treatment on the wood nanostructure (Figure 5). For CMF-treated samples, we observed that increasing the CMF pulse number led to an increase in the mid-q scattering and a shift toward lower q in the diffraction peak. This indicates that multiple pulses of CMF treatment on raw wood promoted a progressive increase in the spacing between cellulose chains as the outer portion of the bundles of EFs was being depolymerized and eroded by CMF oxidation. A similar increase in cellulose chain spacing (in the 200 crystal plane) was also observed previously in brown rot decay (Howell et al., 2011). For decayed samples in our current work, the diffraction peak was not observable in the isotropic curves, which interestingly exhibited nearly identical features to the amorphous curves from the CMF-treated samples.

The similarity between scattering profiles of the decay-treated samples and the CMF-treated samples supports the hypothesis that CMF-treatment causes similar nanostructural changes to those caused by early stage fungal decay. The shift in the diffraction peak indicates that the spacing between elementary fibrils increased as the CMF treatment depolymerized and eroded the outer regions of the cellulose microfibrils as suggested previously in brown rotted wood (Howell et al., 2011). Scattering in the mid-q region also may support the formation of nodules of repolymerized lignin as decay progresses, as has previously been reported in brown rotted wood (Goodell et al., 2017). The overlap observed in the high-q scattering region suggests that the diameter of the cellulose EFs is likely unaffected by the CMF treatment, and we propose that these EFs likely change configuration (i.e., they become more loosely packed) and expand slightly as the cellulose outer layers are also being eroded, supporting an earlier observation in brown rotted wood using X-ray diffraction (Howell et al., 2011). Experiments that probe beyond the q-range achieved in this study would further confirm how CMF modifies the cellulose crystalline structure.

By fitting the data to a model with three structural levels, we quantified the changes caused by the treatments (Table 3). For the CMF-treated samples, changes were observed across the entire q-range. At low-q, the power law exponent decreased with increasing pulses meaning that the treatment led to an increase in the surface roughness of structures that were larger than 200 nm, such as the cellulose microfibril lamellar structure in the S2 cell wall layer and larger re-polymerized lignin particles (Goodell et al., 2017). In the mid-q region, the R_g_ particle size decreased as the CMF-pulse number increased probably reflecting cellulose EFs fragment length, as well as fragments from depolymerized lignin and/or hemicelluloses, while the power law exponents were similar to those from the decay treated samples. For the decay treated samples the most significant changes were observed in the mid-q scattering region, as the low and high-q regions were fixed while fitting the data. For the untreated control sample, scattering from the long EFs gave rise to power law scattering with an exponent of 1. As the samples were decayed, the mid-q power law exponent increased to 2.5, which corresponds to scattering from mass fractals (Beaucage, 1996). The mid-q characteristic dimension R_g_ remained constant beyond 18 days. Scattering in this region has been previously attributed to scattering from lignin aggregates (Pingali et al., 2014; Goodell et al., 2017). Here, we observe that the average size of the aggregates decreased with an increase in CMF pulses. Interestingly, with increasing treatment severity, the mid-q Rg (6 nm) is about double the Rg of spruce lignin monomers in aqueous media reported from computational studies (Vermaas et al., 2019) which we attribute to re-polymerization of the modified lignin in our work. At high-q, changes in the EFs spacing were not detectable in the decay-treated samples, but for the CMF-treated samples an increase of 50% was observed from 1 to 4 pulses.

**TABLE 3:** Comparison between CMF and *G. trabeum* (Gt) decay treatments.

Sample	Low-q	Mid-q		High-q
	*P*_1_	*R*_g_ (nm)	*P*_2_	EF spacing (nm)
UW	3.91 ± 0.16*	–	–	4.66 ± 0.23*
UW-CMF-1	3.72 ± 0.16*	9 ± 1.3	1.6 ± 0.1	5.11 ± 0.17*
UW-CMF-2	3.57 ± 0.21*	7.9 ± 0.5	2.1 ± 0.1	6.40 ± 0.44*
UW-CMF-4	3.40 ± 0.31*	6 ± 1.1	2.5 ± 0.1	7.70 ± 0.15*
0 dGt	4.1 ± 0.07	–	1.2 ± 0.1	5.07 ± 0.24
18 dGt	3.6**	8 ± 2.2	1.8 ± 0.4	7**
42 dGt	3.6**	8 ± 0.7	2.4 ± 0.3	7**

* Parameters were fitted using the anisotropic curve and fixed while fitting the amorphous curve. ** Parameters were fixed while fitting the isotropic curves due to weak features and/or limited q-range.

CMF treatment with an increasing number of pulses was compared to decay by *G. trabeum* over several weeks, and neutron scattering patterns were found to be similar as the severity of treatments increased (Figure 5 and Table 3). Although enzymatic action also likely came into play during decay by *G. trabeum*, the ability of CMF treatment to mimic the degradation of the wood cell wall and achieve similar mid-q characteristic sizes to those generated by brown rot fungal decay is a unique finding. This data suggests that the extracellular CAZymes secreted by *G. trabeum* may largely support CMF action by the fungus in early- to mid-stages of decay, and also supports the finding reported earlier (Goodell et al., 2017) that the function of at least some extracellular CAZymes in the brown rot decay of softwoods is to digest oligosaccharides as they diffuse from the wood cell wall into the lumen space, with limited penetration of enzymes into the wood cell wall. The mechanism may be different in hardwoods as the pattern of attack of the wood cell wall by brown rot fungi has been observed to be different in hardwoods compared to softwoods, with greater cell wall porosity developing in hardwood than in softwood cell walls (Daniel, 2016; Goodell et al., 2017). This suggests a basis for the differential preference of some decay fungi in attacking different woody tissue types (gymnosperm vs. angiosperm) (Riley et al., 2014; Goodell et al., 2017; Krah et al., 2018). *G. trabeum* is a unique brown rot fungus because it has the capacity to also degrade grasses (Kaffenberger and Schilling, 2013; Presley et al., 2018). Prior research has suggested that feruloyl esterases secreted by *G. trabeum* are important in allowing *G. trabeum*, but not the other brown rot fungi included in the analyses, to degrade grasses (Presley et al., 2018). We suggest that the low molecular weight CMF mechanism may also play a role in the selective degradation of grasses by brown rot fungi particularly because enzymes, including feruloyl esterases, would be unable to penetrate deeply in lignified cell walls. CMF factors that may play a role in the selective degradation of grass cell wall material potentially would include: (a) the type of chelator produced by the fungus [hydroquinone/catechol (Paszczynski et al., 1999) vs. variegatic acid (Eastwood et al., 2011) and potentially other chelator structures]; (b) the levels of oxalate produced within the fungal ECM, and the differential pH between the ECM and the plant cell wall; (c) the capacity of the lignin and cellulose in the grass species to bind iron; and (d) the redox capacity of the fungal chelators and cell wall lignin monomers to redox cycle to permit H_2_O_2_ generation within the wood cell wall.

#### Interaction Between the CMF Treatment or Fungal Decay Followed by Enzyme Treatment

Scattering patterns from unmodified wood samples pre-treated solely with a cellulolytic enzymatic cocktail, as expected, did not exhibit any new features, and wood shavings scattered isotropically while disks scattered anisotropically. Both the cloned LMPO and commercial CAZyme enzymatic treatments slightly increased the EF spacing of wood samples following CMF treatment (Table 4). While, it is conceivable that the *G. trabeum* would have a similar effect on the EF spacing, it is not clear from our data if the decay by *G. trabeum* increased EF spacing prior to enzymatic treatment. Because of weak nature of the diffraction peak in the scattering from the wood shavings, the EF values were fixed when fitting those curves (Table 4). We observed that the CMF treatment allowed the cloned LMPO and the commercial CAZyme treatment to further increase spacing of the EFs; however, because of the weak diffraction peak in the wood shaving samples, it remains unclear if decay by *G. trabeum* followed by enzyme treatment also permitted further increase in the EFs. After CMF treatment, LPMO activity demonstrated an equal or better treatment effect compared to that of the commercial CAZyme cocktail, but because comparison of different types of enzymes is difficult based solely on addition of similar amounts of protein, a direct comparison is not possible. These results are important because they show that a recombinant LPMO from a brown rot organism has similar capacity in wood deconstruction compared to commercial enzymes produced by other microorganisms. The findings are also unique in that supplemental enzymatic treatment is able to open the structure of the wood cell wall beyond the levels promoted by CMF treatment. It further suggests that cyclic CMF pretreatment integrated with enzymatic treatment, potentially at low dosages than typically used, may be a more effective way to open the structure of the wood cell wall in biorefinery applications, as opposed to single pretreatments followed by enzymatic treatment.

**TABLE 4:** Comparison between CMF/*G. trabeum* (Gt) decay and enzymes.

Sample	Low-q		Mid-q	High-q
	P_1_	*R*_g_ (nm)	P_2_	EFs spacing (nm)
18 dGt	3.6**	8.02.2	1.80.44	7**
18 dGt-LPMO	3.6**	81.1	1.80.19	7**
18 dGt-CTec2-Htec2	3.6**	71.6	20.5	7**
42 dGt	3.6**	8.00.7	2.40.30	7**
42 dGt-LPMO	3.6**	7.251.38	2.20.54	7**
42 dGt-CTec2-Htec2	3.6**	71.41	2.10.45	7**
UW CMF-1	3.720.16*	91.3	1.60.14	5.110.17*
UW-CMF-1-LPMO	3.720.18*	6.380.69	2.030.31	5.350.29*
UW-CMF-1-CTec2	3.870.22*	91.2	1.560.15	5.300.14*
UW CMF-2	3.570.21*	7.890.48	2.10.13	6.40.44*
UW-CMF-2-LPMO	3.50.2*	6.940.69	2.20.2	7.220.62*
UW-CMF-2-CTec2-Htec2	3.550.12*	7.270.92	2.180.26	6.850.44*

* Parameters were fitted using the anisotropic curve and fixed while fitting the amorphous curve. ** Parameters were fixed while fitting the isotropic curves due to weak features and/or limited q-range.

#### Impact of Pre-delignification on CMF and Enzymatic Treatments

Access of enzymes to wood components has been known to be limited by the presence of recalcitrant lignin. The data above suggest that CMF treatment, and potentially brown rot fungal action, can improve the efficiency of subsequent enzyme action on wood through modification and redistribution of the lignin. This is supported by the similarity of the mid-q scattering features, namely the characteristic size R_g_ and the power law exponent, measured in the CMF-treated samples and the PAA delignified wood samples (Table 5). In the current work, PAA treatment was also used as a reference pretreatment, and specifically for comparison to the ways that lignin was depolymerized/displaced by both the CMF treatment and/or by the enzymatic treatments (the later because of loss of cellulose). It should be noted that the Rg of unmodified Kraft lignin macromolecules in aqueous solution has been observed to be in the range of 40 nm (Sabaghi and Fatehi, 2019), which is similar to the sizes of openings that we observed between elementary fibrils. Moreover, electron microscopy studies on thermochemically treated wood have shown the spacing between microfibrils (bundles of elementary fibrils) to be in the order of 20 nm (Ciesielski et al., 2013), which is similar to the elementary fibril spacing measured in our delignified wood samples. The removal of lignin using PAA was designed to help better distinguish the modes of attack, specifically on cellulose structure. It is conceivable that by delignifying the cell wall with PAA and also by depolymerization of lignin using the CMF treatment, that the regular packing of the elementary fibrils was altered because the constraints of the lignin matrix were removed. This change may have allowed us to observe the naturally polydisperse size of the cellulose microfibrils.

**TABLE 5:** Impact of pre-delignification on CMF and enzymatic treatments.

Sample	Low-q	Mid-q		High-q
	P_1_	*R*_g_ (nm)	P_2_	EFs spacing (nm)
DW	3.5 ± 0.2*	10 ± 1.6	1.9 ± 0.2	16 ± 1.8*
DW-CMF-1	3.65 ± 0.1*	3.6 ± 0.4	2.8 ± 0.4	34 ± 4.6*
DW-CMF-2	3*	5.3 ± 0.4	2.7 ± 0.2	–
DW-LPMO	1.43 ± 0.1 (aligned)	4.5 ± 0.3	2.9 ± 0.2	41.6 ± 19*
	3.1 ± 0.2 (amorphous)			
DW-Ctect2-Htec2	3*	4.4 ± 0.5	2.8 ± 0.4	–
UW	3.9 ± 0.16*	–	–	4.66 ± 0.23*
UW-LPMO	4.11 ± 0.14*	16 ± 4.1	1.3 ± 0.08	4.88 ± 0.12*
UW-CTec2-Htec2	4.59 ± 0.39*	21 ± 4.8	1.5 ± 0.09	4.96 ± 0.11*

* Parameters were fitted using the anisotropic curve and fixed while fitting the amorphous curve.

## Summary/Conclusion

In this research we explored the role that the CMF mechanism plays in the initial decay process by brown rot fungi, “opening up” the wood cell wall structure in advance of enzymatic action. We also explored any potential interaction between CMF constituents and a unique LPMO enzyme. The *G. trabeum*, brown rot LPMO was found to be upregulated early during initiation of brown rot decay, together with genes for a proposed pathway associated with the production of hydroquinones involved in the CMF mechanism.

SANS was used to assess changes in the size and spacing of the nanostructure of wood decayed by the *G. trabeum* and these data were compared to that from wood that was CMF-treated, or CMF-treated wood followed by treatment with either the early-upregulated LPMO or a commercial CAZyme cocktail. PAA delignification treatment, used as a reference, opened the structure of cellulose, and subsequent CMF treatment then further enhanced the effects of the PAA treatment to allow both the CAZyme cocktail and LPMO enzymes to then further increase the spacing between the elementary fibrils.

As expected, neither the LPMO nor the CAZyme cocktail produced structural changes in unmodified wood samples that had not been otherwise pretreated, and the cellulose microfibrils remained unaltered. The CMF treatment of unmodified wood produced neutron scattering patterns similar to that observed in the *G. trabeum* decayed wood, indicating that both brown rot decay and the CMF treatment enlarged the nanopore structure of wood cell walls to permit apparent enzyme access. Even after only a single pulse of CMF treatment, both the LPMO and the commercial CAZyme cocktail enzymes were able to penetrate the cell wall and further increase elementary fibril spacing. Further, our SANS data support that lignin was depolymerized, concurrent with cellulose elementary fibril deconstruction, by CMF action to allow cellulose elementary fibrils to separate, and the lignin was then re-deposited as nanoscale particles (nodules) as previously reported.

Our data suggest that CMF treatment resulted in the oxidation of accessible cellulose fibrils with a concomitant reduction in the size of their cellulose crystallites as attack progressed from the outer regions of cellulose microfibrils. This is similar, and consistent with, the previous scattering data for the pattern of attack observed in brown rotted wood and also similar to prior data on brown rotted wood generated by X-ray diffraction. Multiple pulses of CMF treatment with unmodified wood also produced a progressive increase in elementary fibril spacing, indicating erosion of the cellulose microfibrils by CMF oxidation.

This research confirms earlier work and suggests the potential for the early-upregulated LPMO enzyme from *G. trabeum* to work in association with early-stage CMF chemistry. We propose that *G. trabeum* may have adopted mechanisms to integrate non-enzymatic CMF chemistry and early-upregulated LPMO enzymatic chemistries together during initial stages of brown rot decay.

## Author’s Note

A portion of this research required the use of Neutron scattering instrumentation, and was conducted at the Bio-SANS facility at the High Flux Isotope Reactor (HFIR Project #IPTS-13888); a DOE Office of Science User Facility operated by the Oak Ridge National Laboratory.

## Data Availability Statement

RNA-seq data for this article are deposited at: https://www.ncbi.nlm.nih.gov/geo/query/acc.cgi?acc=GSE108189. The data for the recombinant LPMO enzyme cloned from *Gloeophyllum trabeum* (GtLPMO9A-2) accession number LC157848, is deposited in the DDBJ database: https://www.ddbj.nig.ac.jp/index-e.html. The Small Angle Neutron Scattering (SANS) data can be accessed at: http://doi.org/10.5281/zenodo.3755828.

## Author Contributions

YZ, MY, JJ, and BG developed the experimental design for nanostructural analysis. YZ and MY performed the treatments and SANS analysis with SP and HO’N. BG conceptualized the research, obtained the research support, brought together the research team, and integrated the data analyses. JZ performed the gene expression analysis, metabolite pathway modeling and data workup for the gene expression work, and edited the manuscript. MY and YK provided the information to JZ on properties of the cloned LPMO. SP and HO’N facilitated use and set-up of the SANS beam line, and coordinated with YZ, NP, MY, and BG. NP and SP conducted the bulk of the SANS data analysis with support from YZ. YZ developed the initial draft, and with BG coordinated subsequent drafts. YK and MY provided the LPMO enzyme, input on LPMO use and the write-up on LPMOs. All team members contributed to the final data analysis and writing of the manuscript.

## Conflict of Interest

The authors declare that the research was conducted in the absence of any commercial or financial relationships that could be construed as a potential conflict of interest.
